# How to translate the knowledge of COVID‐19 into the prevention of Omicron variants

**DOI:** 10.1002/ctm2.680

**Published:** 2021-12-12

**Authors:** Xiangdong Wang, Charles A. Powell

**Affiliations:** ^1^ Department of Pulmonary and Critical Care Medicine, Zhongshan Hospital Shanghai Medical College Fudan University Shanghai China; ^2^ Division of Pulmonary, Critical Care and Sleep Medicine Icahn School of Medicine at Mount Sinai New York New York USA

**Keywords:** COVID‐19, disease, Omicron variants, prevention, therapy

## Abstract

Omicron variants are part of the “Coronavirus disease 2019 [COVID‐19] Variants of Concerns” and has the potential to spread around the world rapidly and can harm human life. We can anticipate that the endemic state of COVID‐19 will be characterized by the development of new strains with surges that will predominate in unvaccinated and immunodeficient populations. Thus, there will be an important role in promoting vaccinations, boosters and accessible testing to prevent disease transmission and to rapidly detect surges. There is an urgent need to explore the virology and biology of Omicron variants, define clinical phenomes and therapies, monitor dynamics of genetic changes, and translate the knowledge of COVID‐19 into new variants. Clinical and translational medicine will be impactful in addressing these challenges by providing new insights for understanding and predicting new variants‐associated transmissibility, disease severity, immune escape, diagnostic or therapeutic failure.

Coronavirus disease 2019 (COVID‐19) infection has been ongoing for 2 years, during which COVID‐19‐pandemic patterns, pneumonia and acute respiratory distress syndrome, pulmonary thrombi, clinical phenomes and management strategies have been extensively investigated.^1^, ^2^, ^3^, ^4^ Despite progress in reducing disease fatality and in reducing incidence in regions with high vaccination rates, there remain challenges in confronting the uncertainties introduced by recently identified COVID‐19 variants. Most recently, Omicron (B.1.1.529) was classified and defined as a severe acute respiratory syndrome coronavirus 2 (SARS‐CoV‐2) variant by the Technical Advisory Group on SARS‐CoV‐2 Virus Evolution and announced as one of the “Variant of Concerns” by World Health Organization.^5^ The Omicron (B.1.1.529) variant was first confirmed on 9 November 2021, reported to WHO from South Africa on 24 November 2021, evaluated by the Technical Advisory Group on 26 November 2021, and found in Europe (e.g., the United Kingdom, Germany, Belgium, and Italy) on 27 November 2021. It was predicted that Omicron variants may have spread around the world (e.g., Hong Kong and Israel), although we are still waiting for reports that are limited by inconsistent use of sequencing across the world. Similarly, we await research outcomes to determine if current vaccination platforms can prevent disease and mitigate severity from the Omicron variant. From these ongoing efforts, we will better understand the importance of Omicron following the Delta variant and predict the epidemiological significance of Omicron, acknowledging that the occurrence in South Africa had three distinct peaks in a short period. The Omicron is considered by some to be a high global threat with a risk to human life, although it remains unclear whether the virulence and lethality differ between Omicron and Delta variants and how many people and countries have been or will be infected. The effective prevention and therapeutic managements against Omicron are highly dependent upon the sensitivity and efficiency of the variant surveillance system and infection prevention efforts in each country.

We are facing many unknown issues in preventing and treating Omicron variants. For example, how we can effectively protect ourselves from Omicron and whether the preventive strategy against COVID‐19 still functions to fight Omicron, for example, wearing a mask, washing hands and distancing from others. It is likely that these measures will be effective in mitigating the transmission of Omicron. It is unclear whether the COVID‐19 vaccinations still function in preventing from Omicron variants and whether life‐saving protection and vaccinated populations can be re‐infected, because the Omicron variants may have different numbers and sequencing of mutations that may potentially evade the humoral and cell‐mediated response provided by vaccination. The S gene dropout or S gene target failure as one of the three target genes was not detected in PCR testing of Omicron specimens and this finding is suggested as a potential diagnostic marker for this variant,^5^ though there is an urgent need to completely define the sequence of Omicron variants.

It is of utmost urgency that we define the exact sequences and structures of Omicron variants and validate the accuracy of SARS‐CoV‐2 PCR diagnostics used presently. Because widespread testing will be necessary for mitigating transmission, it will be important to develop a less expensive, point of care antigen testing that will accurately detect Omicron and other relevant variants. National and global vaccinations are considered as one of the most efficient approaches in the prevention of pandemics. Recent data have shown the global impact on cases, hospitalizations and deaths that is provided by vaccination and by boosters. A large number of clinical reports demonstrated that the vaccination of COVID‐19 could prevent the incidence of SARS‐CoV‐2 infection in medium‐ and high‐risk areas of countries.^6^ It is an important and long‐term task to ensure the preventive effects of COVID‐19 vaccinations in the prevention of Omicron and other variants that will follow. The S gene dropout or S gene target failure as one of the three target genes was not detected and suggested as a marker for this variant,^5^ though there is an urgent need to define the sequence of Omicron variants. The information on the exact sequence of those variants will benefit the discovery and development of precious vaccines and diagnostics.

We are questioning how much of our knowledge on COVID‐19 accumulated to date can be generalized for understanding molecular mechanisms and pathogenesis of Omicron variants. For example, inflammatory signatures and machine learning model for accurate classification of COVID‐19 patients with different severity were developed to predict the occurrence of critical illness in patients with COVID‐19.^7^, ^8^ On basis of the systemic inflammatory panel from multiple clinical centres, the exacerbation of disease in COVID‐19 patients could reliably be predicted 20 days before the occurrence. If these approaches can be applied to patients infected with Omicron variants, clinical management can be directed and prioritized to prevent the progression of the disease and optimize resource utilization. Several clinical therapies were developed and validated for COVID‐19 patients with severe illness, such as Dexamethasone, Tocilizumab, convalescent plasma, Chinese traditional medicine, human menstrual blood‐derived mesenchymal stromal cells, and new drugs.^9^, ^10^ The question is how to translate these therapies against COVID‐19 into potential options for patients with Omicron variants.

A challenging issue is the complexity of Omicron variants‐associated evolution and integration since there is a lack of systemic and dynamic investigation on virology and biology of Omicron variants. During the time from the first confirmation of Omicron variant (B.1.1.529) infection on 9 November 2021 to the late report on 30 November 2021, the infection of Omicron variants was confirmed in about 200 people spreading across 30 locations in the world, with uncountable contacted populations. We should pay special attention to the potential interaction of Omicron variants with other viruses, primary and secondary infected bacteria and other pathogens, as well as activated or exhausted immune cells since the virus living and spreading are highly dependent upon the carriers as the host. It is possible that Omicron can be as virulent as Delta or that it may be less virulent, similar to the alpha variant that raised concern early in 2021.

It will be even more complex if the number and location of mutations of Omicron variants dynamically change along with the duration, human races, individuals, and therapy and modify during the replication and binding, to rapidly increase the capacity of infections. We should closely monitor new clusters and functions of circulating immune cells as well as the difference of biological processes and pathways as reported in COVIN‐19 and lung diseases,^11^, ^12^ when we emphasize accelerating the surveillance and sequencing processes of SARS‐CoV‐2 variants. We recently proposed that the single‐cell RNA sequencing of circulating immune cells could be applied as a routine approach of clinical biochemistry to better understand the response of patients with the disease and the role of circulating immune cell involvement.^13^ It would be helpful if we can share those genome sequences and associated metadata to a publicly available database, in order to enhance the capacity of the international community against Omicron variants and improve our knowledge of the SARS‐CoV‐2 Variant of Concerns, for example, epidemiology, severity, the effectiveness of public health and social measures, diagnostic methods, immune responses, antibody neutralization, or other relevant characteristics, as recommended.^5^

In conclusion, Omicron variants are part of the “COVID‐19 Variants of Concerns” and has the potential to spread around the world rapidly and can harm human life. We can anticipate that the endemic state of COVID‐19 will be characterized by the development of new strains with surges that will predominate in unvaccinated and immunodeficient populations. Thus, there will be an important role in promoting vaccinations, boosters and accessible testing to prevent disease transmission and to rapidly detect surges. There is an urgent need to explore the virology and biology of Omicron variants, define clinical phenomes and therapies, monitor dynamics of genetic changes, and translate the knowledge of COVID‐19 into new variants. Clinical and translational medicine will be impactful in addressing these challenges by providing new insights for understanding and predicting new variants‐associated transmissibility, disease severity, immune escape, diagnostic or therapeutic failure.

## References

[1] She J , Jiang J , Ye L , Hu L , Bai C , Song Y . 2019 novel coronavirus of pneumonia in Wuhan, China: emerging attack and management strategies. Clin Transl Med. 2020;9(1):19. 32078069 10.1186/s40169-020-00271-z PMC7033263

[2] Li L , Huang Q , Wang DC , Ingbar DH , Wang X . Acute lung injury in patients with COVID‐19 infection. Clin Transl Med. 2020;10(1):20‐27. 32508022 10.1002/ctm2.16 PMC7240840

[3] Poor HD , Ventetuolo CE , Tolbert T , et al. COVID‐19 critical illness pathophysiology driven by diffuse pulmonary thrombi and pulmonary endothelial dysfunction responsive to thrombolysis. Clin Transl Med. 2020;10(2):e44. 32508062 10.1002/ctm2.44 PMC7288983

[4] Zhang L , Wang DC , Huang Q , Wang X . Significance of clinical phenomes of patients with COVID‐19 infection: a learning from 3795 patients in 80 reports. Clin Transl Med. 2020;10(1):28‐35. 32508041 10.1002/ctm2.17 PMC7240842

[5] WHO . Classification of Omicron (B.1.1.529): SARS‐CoV‐2 variant of concern https://www.who.int/news/item/26‐11‐2021‐classification‐of‐omicron‐(b.1.1.529)‐sars‐cov‐2‐variant‐of‐concern

[6] Xu W , Su S , Jiang S . Ring vaccination of COVID‐19 vaccines in medium‐ and high‐risk areas of countries with low incidence of SARS‐CoV‐2 infection. Clin Transl Med. 2021;11(2):e331. 33634979 10.1002/ctm2.331 PMC7888539

[7] Gao Y , Chen L , Zeng S , Feng X , Chi J , Wang Y , et al. CIRPMC: an online model with simplified inflammatory signature to predict the occurrence of critical illness in patients with COVID‐19. Clin Transl Med. 2020;10(6):e210. 33135353 10.1002/ctm2.210 PMC7577323

[8] Sun C , Bai Y , Chen D , et al. Accurate classification of COVID‐19 patients with different severity via machine learning. Clin Transl Med. 2021;11(3):e323. 33784017 10.1002/ctm2.323 PMC7908044

[9] Jiang W , Li W , Xiong L , et al. Clinical efficacy of convalescent plasma therapy on treating COVID‐19 patients: evidence from matched study and a meta‐analysis. Clin Transl Med. 2020;10(8):e259. 33377664 10.1002/ctm2.259 PMC7752155

[10] Xu X , Jiang W , Chen L , et al. Evaluation of the safety and efficacy of using human menstrual blood‐derived mesenchymal stromal cells in treating severe and critically ill COVID‐19 patients: an exploratory clinical trial. Clin Transl Med. 2021;11(2):e297. 33634996 10.1002/ctm2.297 PMC7839959

[11] Tang H , Gao Y , Li Z , et al. The noncoding and coding transcriptional landscape of the peripheral immune response in patients with COVID‐19. Clin Transl Med. 2020;10(6):e200. 33135345 10.1002/ctm2.200 PMC7548099

[12] Song D , Yan F , Fu H , et al. A cellular census of human peripheral immune cells identifies novel cell states in lung diseases. Clin Transl Med. 2021;11:e579. 34841705 10.1002/ctm2.579 PMC8611783

